# Seroprevalence of *Brucella* spp. and Rift Valley fever virus among slaughterhouse workers in Isiolo County, northern Kenya

**DOI:** 10.1371/journal.pntd.0011677

**Published:** 2023-10-05

**Authors:** Richard Nyamota, Josphat Maina, James Akoko, Daniel Nthiwa, Athman Mwatondo, Mathew Muturi, Lillian Wambua, Earl A. Middlebrook, Andrew W. Bartlow, Jeanne M. Fair, Bernard Bett

**Affiliations:** 1 International Livestock Research Institute, Nairobi, Kenya; 2 Kenya Zoonotic Disease Unit, Ministry of Health and Ministry of Agriculture, Livestock and Fisheries, Nairobi, Kenya; 3 Department of Biological Sciences, University of Embu, Embu, Kenya; 4 Department of Veterinary Medicine, Dahlem Research School of Biomedical Sciences (DRS), Freie Universität Berlin, Berlin, Germany; 5 World Organization for Animal Health, Sub-Regional Representation for Eastern Africa, Nairobi, Kenya; 6 Genomics and Bioanalytic, Los Alamos National Laboratory, Los Alamos, New Mexico, United States of America; Colorado State University, UNITED STATES

## Abstract

*Brucella* spp. and Rift Valley fever virus (RVFV) are classified as priority zoonotic agents in Kenya, based on their public health and socioeconomic impact on the country. Data on the pathogen-specific and co-exposure levels is scarce due to limited active surveillance. This study investigated seroprevalence and co-exposure of *Brucella* spp. and RVFV and associated risk factors among slaughterhouse workers in Isiolo County, northern Kenya. A cross-sectional serosurvey was done in all 19 slaughterhouses in Isiolo County, enrolling 378 participants into the study. The overall seroprevalences for *Brucella* spp. and RVFV were 40.2% (95% CI: 35.2–45.4) and 18.3% (95% CI: 14.5–22.5), respectively while 10.3% (95% CI 7.4%-13.8%) of individuals were positive for antibodies against both *Brucella* spp. and RVFV. Virus neutralisation tests (VNT) confirmed anti-RVFV antibodies in 85% of ELISA-positive samples. Our seroprevalence results were comparable to community-level seroprevalences previously reported in the area. Since most of the study participants were not from livestock-keeping households, our findings attribute most of the detected infections to occupational exposure. The high exposure levels indicate slaughterhouse workers are the most at-risk population and there is need for infection, prevention, and control programs among this high-risk group. This is the first VNT confirmation of virus-neutralising antibodies among slaughterhouse workers in Isiolo County and corroborates reports of the area being a high-risk RVFV area as occasioned by previously reported outbreaks. This necessitates sensitization campaigns to enhance awareness of the risks involved and appropriate mitigation measures.

## Author summary

Brucellosis and Rift Valley fever are zoonotic diseases with significant public health and economic burden in Kenya. We investigated the levels of exposure to *Brucella* spp. and Rift Valley fever virus among slaughterhouse workers in Isiolo County, northern Kenya. We found high exposure levels that are comparable to those previously reported within the community. Occupational exposure was the most probable source of infection since most of the workers did not keep livestock. Therefore, slaughterhouses could be targeted for sensitization on appropriate infection control measures besides being important points for disease surveillance.

## Introduction

Slaughterhouse workers face many occupational health hazards including physical, chemical and biological hazards that are primarily from zoonotic pathogens [1,2]. Slaughterhouse workers may be exposed to zoonotic pathogens from handling potentially infected animal tissues and, inhalation of aerosolized infectious droplets [3,4]. Often, the risk of exposure is exacerbated by a lack of personal protective equipment (PPE) [4,5].

Brucellosis and Rift Valley fever (RVF) are two of the most common occupational zoonoses that slaughterhouse workers are exposed to in the sub-Saharan Africa [6–11]. In Kenya, the two diseases are ranked among the top five zoonoses due to their significant public health burden and associated economic impacts [12]. Brucellosis is endemic in most arid and semi-arid regions of Kenya, while RVF is an epidemic-prone disease that is associated with cyclic outbreaks every five to ten years in the country [13–18].

The two diseases present in a wide spectrum of syndromes in humans, posing significant challenges in their diagnosis, especially in remote, resource poor-settings where they are most prevalent [19,20]. Human cases of RVF might present clinically as febrile illnesses or as serious disorders including encephalitis, hepatitis, or retinitis [19,21]. Brucellosis mainly presents as an undulating fever with varying constitutional symptoms [22,23]. Both diseases are primarily transmitted to humans through contact with tissues from infected animals; this explains why slaughterhouse workers are classified into the most at-risk group of people [24,25]. The magnitude of zoonotic transmission is correlated with several factors including the disease endemicity in livestock populations, animal husbandry systems, hygiene, and forms of animal product consumption [26–28]. In most agrarian communities, animals with abortion symptoms, one of the main clinical presentations of infection with the two pathogens, end up being slaughtered owing to their reduced productivity [29]. This presents a significant risk of exposure among slaughterhouse workers.

This study investigated the seroprevalence of *Brucella* spp. and RVFV and associated risk factors among slaughterhouse workers in Isiolo County in northeastern Kenya. Findings from the study would inform the development of prevention and control measures among the slaughterhouse workers in the area.

## Materials and methods

### Ethical statement

Ethical clearance was obtained from the International Livestock Research Institute (ILRI) Institutional Research Ethics Committee (IREC), reference ILRI-IREC2021-24. The study background and objectives were explained to the study participants before written consent was obtained.

### Study area

The study was conducted in Isiolo County, a low-lying arid and semi-arid region of northern Kenya. The area has a mean elevation of 1,145m above sea level and receives sparse annual rainfall amounting to 150–250 mm [30]. Isiolo County is sparsely populated with a population of 268,000 residents who mostly reside in rural areas [31]. Nomadic pastoralism is the main source of livelihoods in the area, with at-least 90% of residents directly depending on livestock for sustenance and economic wellbeing [32]. Isiolo also has game parks and reserves with significant populations of free-roaming wildlife.

### Study population

The study population was slaughterhouse workers from all wards (administrative regions) of Isiolo County. Slaughterhouses were defined as establishments where livestock animals (cattle, camels, sheep, and goats) are slaughtered for consumption, and individuals working here were considered slaughterhouse workers [33]. Inclusion criteria were all slaughter facilities where animals are commercially slaughtered, ranging from small-scale slaughter slabs with limited mechanization to well-equipped facilities with advanced meat processing capabilities [34].

### Sample size estimation

This study used a cross-sectional sampling approach where slaughterhouses and workers were considered as primary and secondary sampling units respectively. The minimum sample size (n) was derived using the Krejcie and Morgan formula for cross-sectional studies with finite population *n* = χ^2^NP(1-p)+d^2^(N-1)+ χ^2^(1-P) with a precision level (d) set at 0.05 and χ^2^ at 3.841 (table value of chi-square for 1 degree of freedom at the desired confidence level) [35]. A priori prevalence (P) of 50% was assumed for both *Brucella* sp. and RVFV and a population size (N) of 1000 which gave a minimum sample size of 278.

### Sampling strategy and sample collection

**Fig 1:**
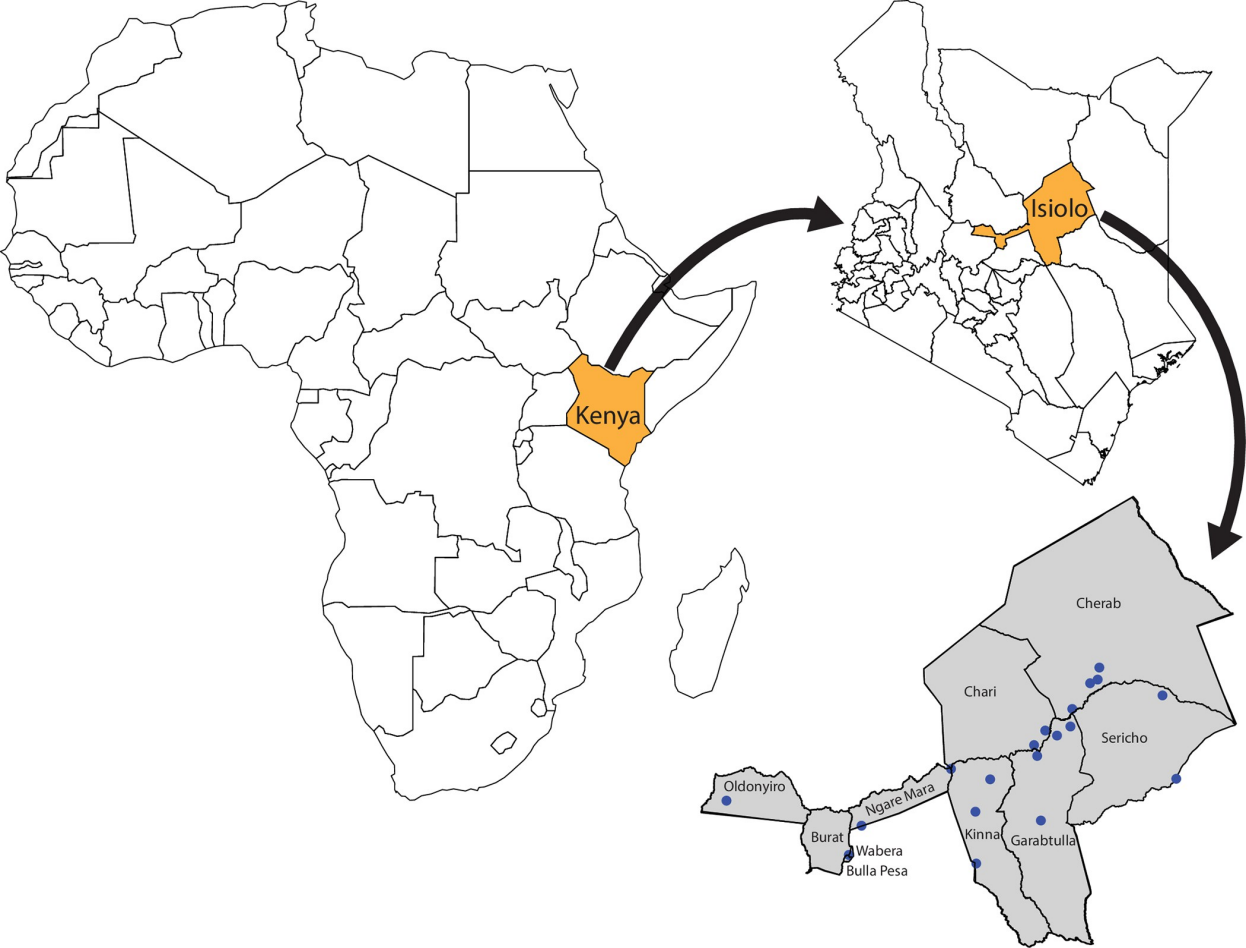
A continental map of Africa highlighting the location of Kenya (Orange) with a blown-up map of Kenya showing the location of Isiolo County (orange) and a magnified map of Isiolo County (grey) showing the locations of slaughterhouses that were used in the study (blue dots). The base map of Africa was created in R using the function ’wrld_simpl’ (Region 2) in the maptools package [36]. The base map shapefile for Africa was downloaded from: Natural Earth (http://www.naturalearthdata.com/). The Kenya counties shapefile was downloaded from: https://data.amerigeoss.org/dataset/47-counties-of-kenya/resource/11e8697c-48a9-4af5-85f9-f5a2b0625d2c. This work is licensed under a Creative Commons Attribution [4.0] License. The wards shapefile was downloaded from: https://data.amerigeoss.org/dataset/kenya-admin-level-3-wards/resource/2fe6fcaa-58af-4d5d-a17d-81c73574ae2a. This work is licensed under a Creative Commons Attribution [4.0] License.

All 19 slaughterhouses in various administrative wards in Isiolo County were identified and sampled during the dry season between 26^th^ November to 2^nd^ December 2021 (Fig 1). The slaughterhouses in this area were visited to determine the number of workers per slaughterhouse before sampling. Everyone who worked in a slaughterhouse at the time of sampling (work was defined as slaughter, butchering, flaying and any form of handling animal carcasses, slaughterhouse owners, transporters, inspectors, security guards, and cleaners) and gave consent for sampling and data collection was included in the study. The selected participants responded to questionnaires before being sampled. The data collected on the questionnaire included slaughterhouse name and its geographic coordinates, socio-demographic information (gender, marital status, education level, occupation, and age), physiologic parameters (body temperature, blood pressure, pulse rate, height, weight), animal husbandry practices, eating habits, occupational hazards encountered, occupational roles and occupational hygiene practices (use of personal protective equipment, cut hands, fluid splash).

Blood samples were collected through venous puncture by a certified clinician. Sera samples were collected in 4 ml plain vacutainers and barcoded. Sample barcodes and questionnaire data were collected using electronic forms designed with open data kit (ODK) application and data was uploaded to a centralized cloud-based server at International Livestock Research Institute (ILRI). The samples were processed for serum in the same day of collection at the field laboratory and stored at -20°C, after which they were transferred to the ILRI laboratory in Nairobi for further testing.

### Laboratory assays

Enzyme-linked immunosorbent assay (ELISA) was used to screen the serum samples for anti-RVFV and anti-*Brucella* antibodies using validated commercial kits. Testing for RVFV was done using ID Screen RVFV competition multispecies ELISA kit (ID VET, Montpellier, France) [37,38], whereas *Brucella* assays were done using IBL-America IgG-ELISA kits (Minneapolis, MN) [39–41]. Both kits have been shown to have specificity above 98%, whereas the sensitivity of the RVFV and *Brucella* kits was 85% and 100%, respectively [42,43]. All assays were done as described by the manufacturers. The optical densities were recorded using a Synergy BioTek microplate ELISA reader (Synergy, BioTek, Winooski, VT, USA) at 405 nm for *Brucella* and 450 nm for RVFV. Seropositive samples for RVFV were subjected to Virus Neutralization Tests (VNT) [44] to confirm the presence of anti-RVFV antibodies. Briefly, the serum samples were heat inactivated at 56°C for 30 min. Two-fold serial dilution of the serum was then done in Dulbecco’s Modified Eagle Medium (DMEM) supplemented with 10% fetal bovine serum (FBS). The live attenuated Smithburn RVFV vaccine strain clone 13 (CL13T) was added into the diluted serum at 100 TCID_50_ per well and incubated at room temperature for 1 hour. This virus-serum mixture was added onto a confluent monolayer vero cells prepared at 2x10^4^ cells/well and incubated at 37°C and 5% CO^2^ for three days_._ Afterwards, the cells were observed under an inverted microscope for cytopathic effect (CPE). Where CPE was not observed, the serum samples were considered to have virus-neutralizing antibodies and vice versa.

### Statistical analysis

All the data analyses were done using R statistical software, version 4.22 [45]. The *Brucella* spp. and RVFV seroprevalence estimates and their 95% confidence intervals (CI) were estimated using *binconf* function within the *Hmisc* package [46]. Exploratory analyses were done using the seropositivity status for each disease and the demographic factors as dependent and independent variables, respectively. The associations between dependent and independent variables were assessed by chi-square tests at a significance level of α = 0.05 level and further using univariable models. Multivariable analysis using outcome variables for each disease was done on independent variables whose univariable results had p-values of ≤0.2. Both univariable and multivariable analyses were done using the generalized linear mixed models with logit link functions. These were implemented in R using the *glmer* function within the *lme4* package [47]. In these models, we fitted slaughterhouse as a random effects variable to account for the potential clustering of observations at this level. The significance of the fixed effects variable was evaluated using the Likelihood ratio test (at α = 0.05). For multivariable analysis, a backward selection procedure was used to build a parsimonious model from a full model that included all the variables that met the inclusion criterion (p≤0.2) mentioned above. The best-fitting model was determined by comparing all the possible maximal, minimal and null-intercept-only models using analysis of variance (ANOVA). The final model that gave the lowest Akaike information criterion (AIC) was selected. We extracted the variance estimates for random effects from the final models for each disease using the *varCorr* function in *lme4* package. The intra-class correlation coefficients (ICCs) were estimated from these models using the *icc* function in *performance* package [48]. Finally, we also calculated the 95% confidence intervals (CI) for the variance and ICC estimates via bootstrap simulation.

## Results

### Subject characteristics

We sampled 378 slaughterhouse workers comprising 136 (35.9%) females and 242 (64.0%) males from 19 slaughterhouses. The median number of workers sampled per slaughterhouse was 10 (range 2–150); their ages ranged from 18–75 years with a mean of 38.7 (95% CI 37.3–40.2). Eight years (range 0.1–50.1) was the average length of time spent working as a slaughterhouse worker. Of the recruited subjects, 39.2% primarily slaughtered and dressed carcasses, 33.1% were slaughterhouse owners, 19.6% were transporters, 2.9% were inspectors, while the remaining 5.3% classified as “others” comprised security guards, and cleaners (Table 1).

**Table 1:** Sample demographics and descriptive analysis for *Brucella* spp. and RVFV seroprevalence estimates amongst slaughterhouse workers in Isiolo county. The test for statistical significance was done at α = 0.05.

		*Brucella spp*.				RVFV			
Variable	Level	positive(n)	Seroprevalence% (CI)	χ^2^	*P*	positive	Seroprevalence% (CI)	χ^2^	*P*
Education	No education	57(156)	36.5% (29.0–44.6)	3	0.39	35	22.4% (16.2–29.8)	3.6	0.3
	Primary	50(126)	39.7% (31.1–48.8)			20	15.9% (10.0–23.4)		
	Secondary	34(75)	45.3% (33.8–57.3)			12	16.0% (8.6–26.3)		
	Tertiary	11(21)	52.4% (29.8–74.3)			2	9.5% (1.2–30.4)		
Ward	Bullapesa	50(19)	38.0%(24.7–52.8)	7.48	0.59	7	14.0%(5.8–26.7)	7.10	0.63
	Burat	89(37)	41.6%(31.2–52.5)			15	16.9%(9.8–26.3)		
	Chari	21(12)	57.1%(34.0–78.2)			5	23.8%(8.2–47.2)		
	Cherab	53(18)	34.0%(21.5–48.3)			12	22.6%(12.3–36.2		
	Garbatulla	40(19)	47.5%(31.5–63.9)			9	22.5%(10.8–38.5)		
	Kinna	39(12)	30.8%(17.0–47.6)			8	20.5%(9.3–36.5)		
	Ngaremara	11(6)	54.5%(23.4–83.3)			3	27.3%(6.0–61.0)		
	Oldonyiro	23(10)	43.5%(23.2–65.5)			2	8.7%(1.1–28.0)		
	Sericho	43(15)	34.9%(21.0–50.9)			5	11.6%(3.9–25.1)		
	Wabera	9(4)	44.4%(13.7–78.8)			3	33.3%(7.5–70.1)		
Sex	Female	49(136)	36.0% (28.0–44.7)	1.3	0.26	29	21.3% (14.8–29.2)	1	0.31
	Male	103(242)	42.6% (36.3–49.1)			40	16.5% (12.1–21.8)		
Trained (Job related)	No	122(313)	39.0% (33.5–44.6)	0.9	0.35	55	17.6% (13.5–22.3)	0.3	0.56
	Yes	30(65)	46.2% (33.7–59.0)			14	21.5% (12.3–33.5)		
Primary role	Slaughtermen	26(148)	17.6% (11.8–24.7)	6.8	0.15	54	36.5% (28.7–44.8)	4.5	0.35
	Inspector	4(11)	36.4% (10.9–69.2)			4	36.4% (10.9–69.2)		
	Other (Cleaner/Security guards)	3(20)	15.0% (3.2–37.9)			12	60.0% (36.0–80.9)		
	Owner	28(125)	22.4% (15.4–30.7)			50	40.0% (31.3–49.1)		
	Transporter	8(74)	10.8% (4.8–20.2)			32	43.2% (31.8–55.3)		
Wear PPE	No	82(213)	38.5% (31.9–45.4)	0.4	0.51	47	22.1% (16.7–28.2)	4.2	0.04
	Yes	70(165)	42.4% (34.8–50.4)			22	13.3% (8.6–19.5)		

### Descriptive factors information

Despite most workers encountering fluid splash and 3% having open wounds, only a small proportion of them used personal protective equipment (PPE). Gumboots were the most frequently used PPE (33.6%) followed by aprons (27%) while a limited number used hand gloves (6.4%) and eye protection equipment (1.3%). Interestingly, handwashing was commonly practiced (78.0%) within the occupational environment. Most of the slaughterhouse workers had no formal education and lacked job-related training. A small proportion of the respondents 12.4% (47/378) owned livestock and a third of all the respondents undertook various animal husbandry roles that included cleaning, milking, and assisting in animal birthing besides working in the slaughterhouses. Over 96.0% of interviewed respondents consumed both meat and milk. Among these, 12.7% reported routine consumption of raw meat while none confirmed whether they consumed raw milk. More than 30.0% of the respondents reported to have had contact with ill animals and aborted material in the last three months. Finally, arthropod bites were reported by 28% of the respondents.

### *Brucella* spp., and RVFV seroprevalence and associated risk factors

The overall seroprevalence for *Brucella* spp. and RVFV was 40.2%, (95% CI 35.2–45.4) and 18.3% (95% CI 14.5–22.5), respectively. Out of the 69 samples that were positive for RVFV by ELISA, 59 were confirmed to have anti-RVFV antibodies by VNT. There was no significant difference in RVFV seropositivity between male (16.5%, 95% CI 12.1–21.8) and female workers (21.3% 95% CI 14.8–29.2) χ^2^ = 1.0, p-value = 0.30). Likewise, the *Brucella* spp. seroprevalence between male (42.6%, 95%CI 36–49) and female (36.0%, 95%CI 28–45) workers did not vary significantly (χ^2^ = 1.3, p = 0.30). Moreover, 10.3% (95% CI 7.4%-13.8%) of the individuals tested positive for both anti-*Brucella* spp. and anti-RVFV antibodies. Arthropod bites did not significantly affect RVFV seropositivity (χ^2^ = 0.00, p = 0.96). The results for the association between various independent variables for *Brucella* spp. and RVFV seroprevalence are also presented in Table 1. However, we also observed a significantly higher seropositivity of RVFV among individuals who did not use PPE compared to those who used them (χ^2^ = 4.18, p = 0.04). This was also observed for co-exposure with both diseases although not statistically significant (χ^2^ = 1.44, p = 0.23). None of the univariable and multivariable analyses were statistically associated with either *Brucella* spp. or RVFV seropositivity.

## Discussion

This study reports the seroprevalence and co-exposure of *Brucella* spp. and RVFV and related risk factors amongst slaughterhouse workers from pastoral communities in Isiolo county. Interestingly, most of the respondents in our study were not from livestock-keeping households and thus exposure to *Brucella* spp. and RVFV is likely to be occupational. This corroborates previous studies that have reported Isiolo to be a high-risk area for *Brucella* spp. and RVFV infections. Our findings on age and sex concur with previous human demographics reports from slaughterhouse workers [9,10,49,50]. This implies young male workers to be the more susceptible group that commonly work in slaughterhouses. However, there was no association between subject-level variable and either *Brucella* spp. or RVFV seropositivity [10]. This could be attributed to the fact that the slaughterhouse workers recruited for the study carried out multiple chores ranging from carcass dressing, evisceration, slaughtering and cleaning and hence they had similar levels of exposure. We also hypothesize that the inadequate biosafety and biosecurity measures that were being practised in the facilities presented multiple pathways through which most of the workers, irrespective of their personal characteristics and assigned chores, got exposed to the pathogens of interest.

The observed *Brucella* spp. and RVFV seroprevalence among slaughterhouse workers was comparable to previous community-level findings from the same region [15,51]. However, our findings on the seropositivity to both infections was not associated to the frequently mentioned risk factors, such as eating patterns and animal husbandry techniques [18,52]. Our findings are also comparable to other studies from Africa that did not find significant association for the risk factor variables and seropositivity for RVFV and *Brucella* in humans [9,37]. This could be attributed to the finite human populations sampled, the common and multiple exposures across the slaughter facilities where individuals routinely undertake multiple roles. This plausibly makes it difficult to correlate seropositivity to individual risk factors. Consistent with data from Tanzania, our findings shows that most workers were from non-livestock-keeping households, thereby suggesting occupational exposure within the slaughterhouses [50]. The overall co-exposure to both *Brucella* spp. and RVFV concurs with previous studies that reported co-exposure as a natural occurrence in livestock and wildlife in Kenya [14,53]. This implies possible human co-exposure to zoonotic pathogens with common clinical presentations which may lead to incomplete diagnosis of clinical syndromes. Detection of RVFV-neutralizing antibodies in a large proportion of the ELISA-positive individuals confirms human exposure to the virus in this region thereby corroborating the status of slaughterhouse workers as a high-risk group. Further investigations including the RVFV incidence within slaughter facilities would be beneficial in establishing the disease burden, strengthening the surveillance system, and assessing the suitability of slaughterhouse sampling sites in predicting RVFV epidemic cycles.

Failure to use PPE and performing multiple duties at a slaughterhouse were risk factors related with RVFV seropositivity, although not statistically significant. Some respondents also undertook other roles including animal husbandry, such as animal birthing, which could facilitate the transmission of zoonotic diseases. Our findings thus emphasize the significance of sensitizing slaughterhouse workers, considered a high-risk group, about infection control measures such as the use of appropriate PPE as recommended in previous studies [54,55]. Gumboots were the most often worn PPE in Isiolo, as reported in another study from Ugandan, however their effectiveness against *Brucella* spp. and RVFV could not be determined [56].

Inspectors and owners of the slaughterhouses had the highest exposure levels for RVFV, although this observation was not statistically significant. The plausible cause for this observation would be the numerous carcases the inspectors were expected to handle each day, thereby elevating their exposure to potentially infectious material. Despite more than 90% of the respondents consuming both milk and meat, this was not significantly associated with either *Brucella* or RVFV seropositivity. This suggests limited exposure to the infectious pathogen through this route for the slaughterhouse workers, this could be attributed to reported low prevalence of *Brucella* in milk [18]. This further corroborates our hypothesis of likely occupational exposure at the slaughterhouse since most sampled workers did not keep livestock. However, our results should be cautiously interpreted due to the skewed distribution of the individuals who consumed milk and meat (90%) with those who did not (10%) that may affect the statistical power to compare the two groups.

Our study, however, had a few limitations. First, subjects were self-selected at each slaughterhouse because only individuals that provided informed consent for the participation in the study were sampled. Ideally, a random process to select these subjects would have been used to obtain a representative study population and avoid biases due to self-selection. Secondly, the cross-sectional study design and the types of ELISA kits used (IgG) do not allow for the determination of new infections. It is therefore likely that cases observed included a large proportion of historical exposures which might have been acquired in other locations. Additionally, we lacked data on RVFV vector exposure in the research area and thus could not account for its effect on the observed RVFV seropositivity. Although the study focussed on humans, future ante- and post-mortem inspections of animals and collection of samples can also generate a lot of data for animal bio-surveillance.

In conclusion, we demonstrate that slaughterhouse workers in Isiolo County suffered relatively high levels of exposure to *Brucella* and RVFV mostly through routine occupational activities. Secondly, clinical cases observed among slaughterhouse workers should be urgently addressed since they could signify a bigger health challenge at the community level. To address the zoonotic diseases burden through a one-health approach, the government of Kenya established the zoonotic diseases unit (ZDU). One of the outputs has been the prioritization of key zoonotic diseases in Kenya [12]. However, data paucity owing to limited sustainable surveillance and diagnostic capacity in remote areas is a significant challenge to the implementation of comprehensive disease control measures in Kenya [57]. Given the high risk of infectious diseases in abattoir environments, our study demonstrates that slaughterhouses and slaughterhouse workers can be critical sentinel surveillance sites for priority zoonotic pathogens at the community level.

## Supporting information

S1 Data Data that underlies this paper. (CSV)
